# Supplementary figures and images for: Genome-Wide Analysis Reveals Changes in Long Noncoding RNAs in the Differentiation of Canine BMSCs into Insulin-Producing Cells
Source: Int J Mol Sci. 2020 Aug 3;21(15):5549. doi: 10.3390/ijms21155549 (PMC7432238; doi:10.3390/ijms21155549)

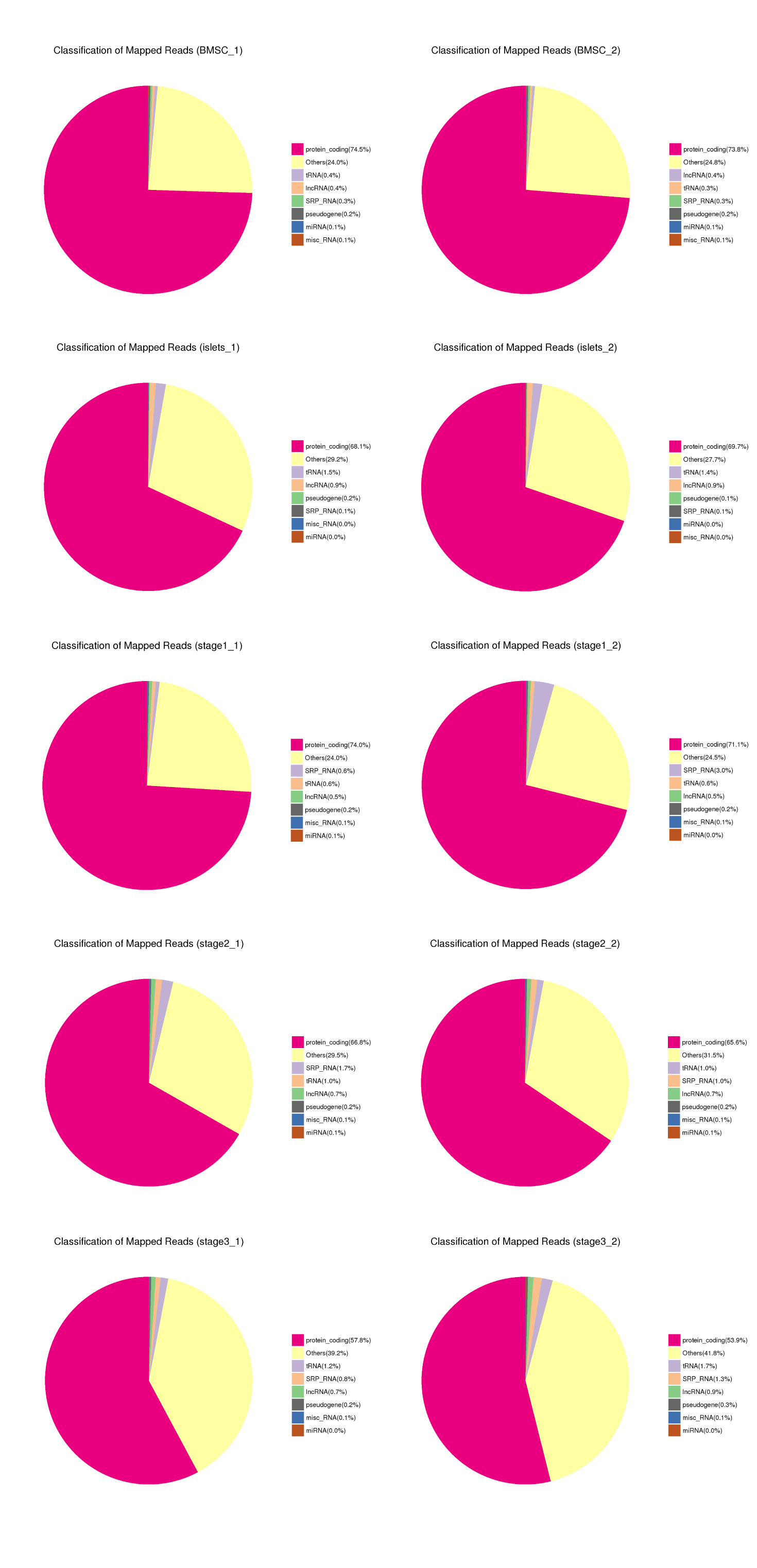

Supplement: Supplementary file 1 [file ijms-21-05549-s001.zip › supplementary figure1.tif]
